# Supplementary material for: Longitudinal Dynamics of SARS-CoV-2 IgG Antibody Responses after the Two-Dose Regimen of BNT162b2 Vaccination and the Effect of a Third Dose on Healthcare Workers in Japan
Source: Vaccines (Basel). 2022 May 24;10(6):830. doi: 10.3390/vaccines10060830 (PMC9229433; doi:10.3390/vaccines10060830)
Supplement: Supplementary file 1 [file vaccines-10-00830-s001.zip › vaccines-1706767-supplementary.pdf]

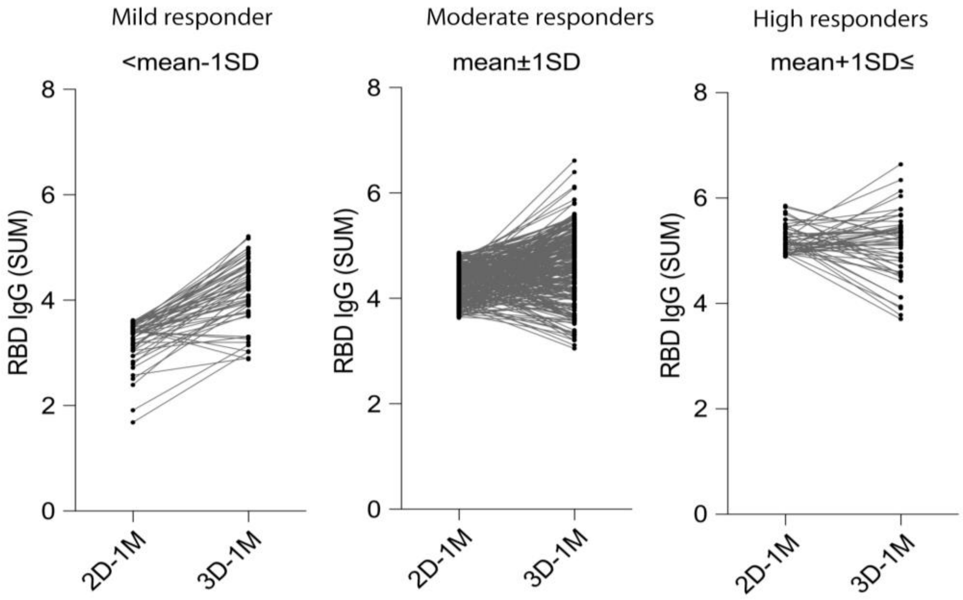

Figure S1. Anti-RBD IgG titers at 1M after the second dose and third dose of BNT162b2 vaccination of Mild responder (< mean -1SD), Moderate responders (mean ± 1SD) and High responders (mean + 1SD≤). Cohort participant were divided into three groups based on anti-RBD IgG antibody titers at 2D-1M as shown in Figure 5A.

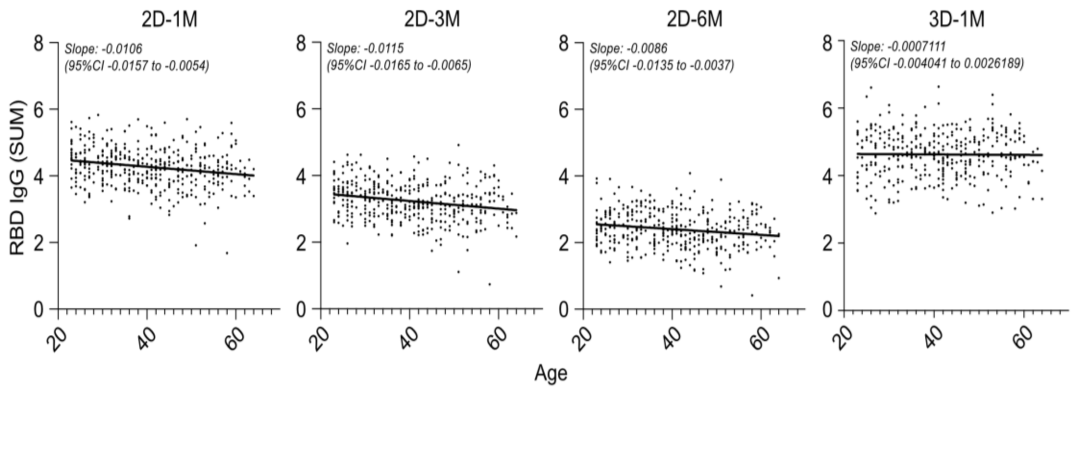

Figure S2. Regression analysis of anti-RBD IgG titers with age of individuals obtained at 2D-1M, 2D-3M, 2D-6M and 3D-1M. Linear regression lines are shown in black solid lines.

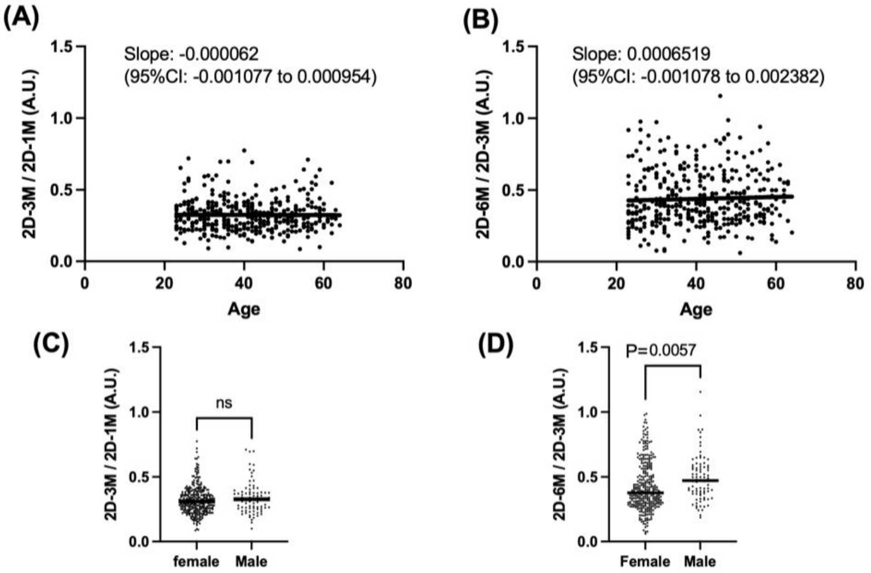

Figure S3. Longitudinal kinetics of Anti-SARS-CoV-2 RBD IgG antibodies after second dose of BNT162b2 vaccine by age and sex. (A, B) Linear regression analysis on the effect of age on the attenuation of anti-SARS-CoV-2 RBD IgG titers (A.U.) between 2D-1M and 2D-3M (A) and between 2D-3M and 2D-6M(B). The 2D-3M to 2D-1M and 2D-6M to 2D-3M antibody titers in each individual are indicated, respectively. (n=383) (C, D) Effect of gender difference in the attenuation of anti-SARS-CoV-2 RBD IgG titers(A.U.) between 2D-1M and 2D-3M(C) and between 2D-1M and 2D-6M (D). The 2D-3M to 2D-1M and 2D-6M to 2D-3M antibody titers in each individual are indicated, respectively. (n= 383) 95%CI: 95% confidence interval. ns: not significant.

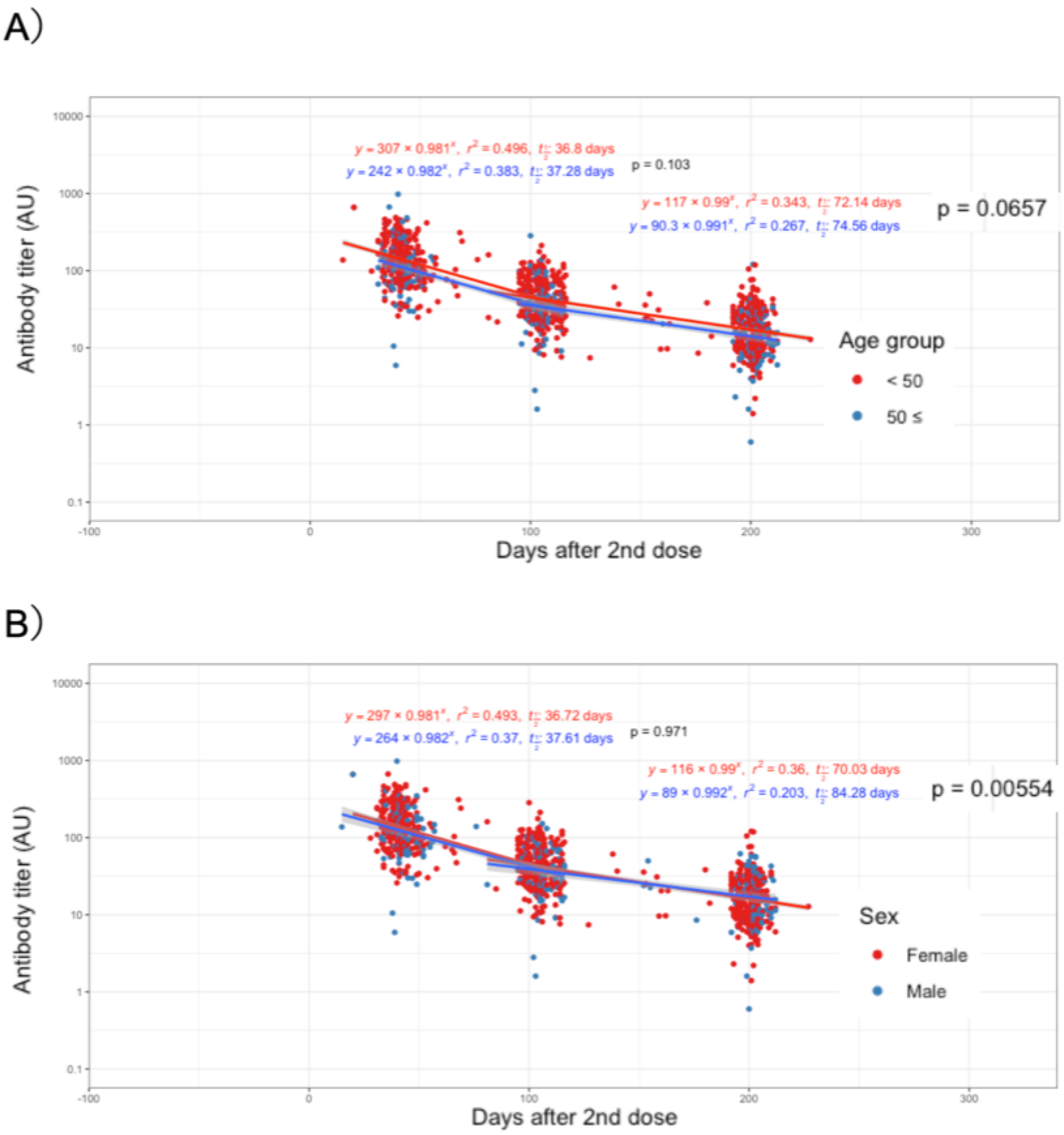

Figure S4. Longitudinal kinetics of Anti-SARS-CoV-2 RBD IgG antibodies after the two doses of BNT162b2 vaccine. Linear regression analysis of total of 1252 samples of post-vaccination from 431 subjects grouped by age (A: less than 50 or more than 50) and sex (B).
